# Real-time single molecular study of a pretreated cellulose hydrolysis mode and individual enzyme movement

**DOI:** 10.1186/s13068-016-0498-x

**Published:** 2016-04-12

**Authors:** Yanan Zhang, Mengmeng Zhang, R. Alexander Reese, Haiqian Zhang, Bingqian Xu

**Affiliations:** Single Molecule Study Laboratory, College of Engineering and Nanoscale Science and Engineering Center, University of Georgia, Athens, GA 30602 USA; College of Materials Science and Technology, Nanjing University of Aeronautics and Astronautics, Nanjing, 210016 People’s Republic of China

**Keywords:** Pretreated plant cell wall, AFM recognition imaging, Real-time, Single-molecule, Hydrolysis mode

## Abstract

**Background:**

The main challenges of large-scale biochemical conversion involve the high costs of cellulolytic enzymes and the inefficiency in enzymatic deconstruction of polysaccharides embedded in the complex structure of the plant cell wall, leading to ongoing interests in studying the predominant mode of enzymatic hydrolysis. In this study, complete enzymatic hydrolysis of pretreated biomass substrates was visualized in situ and in real time by atomic force microscopy (AFM) topography and recognition imaging. Throughout the entire hydrolytic process, a hydrolysis mode for exoglucanase (CBH I) consisting of a peeling action, wherein cellulose microfibrils are peeled from sites on the pretreated cellulose substrate that have cracks sufficiently large for CBH I to immobilize.

**Results:**

We quantitatively monitored the complete hydrolytic process on pretreated cellulose. The synergetic effect among the different enzymes can accelerate the cellulose hydrolysis rate dramatically. However, the combination of CBH I and β-glucosidases (β-G) exhibited a similar degradation capacity as did whole enzyme (contains the cellobiohydrolases and endoglucanase as its major enzyme components). We developed a comprehensive dynamic analysis for individual cellulase acting on single pretreated cellulose through use of functional AFM topography and recognition imaging. The single crystalline cellulose was divided into different regions based on the cracks on the substrate surface and was observed to either depolymerize or to peel away by the jammed enzyme molecules. After the exfoliation of one region, new cracks were produced for the enzyme molecules to immobilize. The fiber width may have a relationship with the peeling mode of the fibers. We performed a statistical height measure of the generated peaks of the peeled fibers. The height values range from 11 to 24 nm. We assume that the CBH I enzymes stop progressing along the cellulose microfibril when the peeled microfibril height exceeds 11 nm.

**Conclusion:**

The combination of CBH I and β-G can achieve an effective hydrolysis of the pretreated biomass substrates. The single-molecule study of the complete hydrolytic process indicates that the hydrolytic mode involves the peeling of the microfibrils and progressive depolymerization, which depend on the size of the cracks on the surface of the pretreated cellulose microfibrils.

**Electronic supplementary material:**

The online version of this article (doi:10.1186/s13068-016-0498-x) contains supplementary material, which is available to authorized users.

## Background

The production of ethanol and other biofuels from cellulosic biomass has garnered significant focus as global energy demands have risen, leading to the increased use of fossil fuels which exist in a finite quantity and contribute to climate change [1]. The main challenges of large-scale biochemical conversion of biomass into biofuel are the high costs of cellulolytic enzymes and the inefficiency in enzymatic deconstruction of polysaccharides embedded in the complex structure of the plant cell walls [2]. In order to boost hydrolytic efficiency and reduce costs, pretreatment is usually required to disrupt the bonding between cellulose and both soluble hemicellulose and lignin to reduce the recalcitrance of cell walls and ease the access of cellulolytic enzymes [3]. Multiple integrated pretreatment steps have been reported, specifically by steam explosion or dilute acid pretreatment followed by a delignification step. This pretreated biomass may consist of up to 90 % cellulose and therefore significantly facilitate enzymatic hydrolysis [4]. Enzymatic hydrolysis of cellulose to glucose is one of the major steps involved in the conversion of cellulosic biomass to yield biofuel. Currently, the enzymes that can make near-complete use of plant cell walls for biofuel conversion are generally obtained from industrial fermentation of an important fungus, *Trichoderma reesei* [5]. Exoglucanase (CBH I) accounts for over 60 % of the enzymes prepared from *T. reesei* [6]. Such CBH I which is also known as the “processive” cellulase appears to catalyze most bond-cleavages in the saccharification of crystalline cellulose [7]. Processive hydrolysis consists of adsorption of the carbohydrate-binding module (CBM) domain to the cellulose surface and complexation of the catalytic domain to a cellulose chain. Cellulose is efficiently hydrolyzed by synergetic action of CBH and endoglucanases (EG).

In order to improve the performance of cellulases, the mechanism of action of these enzymes on the surface of cellulose must be understood at the molecular level [8]. Recently, there has been a significant increase in the number of studies on the dynamics of the enzymatic hydrolysis of cellulose. Some theoretical models present various factors affecting the enzymatic rates and activities, while the exact mechanism of cellulases on biomass remains incompletely understood. The contributing factors decreasing hydrolysis rates in the models that have been studied include enzyme deactivation, biphasic composition of cellulose, decrease in substrate reactivity and accessibility, jamming and fractal kinetics, and a decrease in the synergism [9]. It is difficult to conclude which of the limitations are predominant from the models when each factor was investigated separately and thoroughly. Comprehensive and intuitive experimental data are much necessary to get insight into hydrolysis process. There have been extensive investigations on the mechanism of hydrolysis of cellulose fibers using different technologies. Nieves et al. [10] and Fox et al. [11] directly studied the cellobiohydrolase complexation kinetics using a high-performance liquid chromatography (HPLC) system. Komanoya et al. [12] characterized the cellulose hydrolysis process by physicochemical methods such as x-ray diffraction (XRD), transmission electron microscope (TEM), x-ray photoelectron spectroscopy (XPS), H_2_-temperature programming reduction (H_2_-TPR), O_2_-titration, and x-ray absorption fine structure (XAFS). Atomic force microscopy (AFM) is overall a more comprehensive alternative method that provides high-quality images of the cellulose samples with nanometer resolution and allows in situ, real-time degradation to be observed [13, 14]. Quirk et al. [15] demonstrated the tremendous potential of AFM in studying the mechanism of enzymatic digestion of cellulose and identifying the most effective enzymes in degrading various cellulose structures. Recently, researchers successfully employed high-speed atomic force microscopy (HS-AFM) to investigate the action of the enzyme molecules on crystalline cellulose, allowing real-time, dynamic visualization of structural changes during the hydrolytic process. According to previous research, in which the crystallinity remained unchanged and the surface roughness increased, the hydrolysis of cellulose is supposed to take place in the outer layer of the substrate surface and to proceed layer by layer [16]. It has been suggested that the enzymatic hydrolysis of pure cellulose by CBH I could occur in two ways: cellulose fibrils are peeled off the external surface or are depolymerized by the cellulase mixture entering pores and fissures large enough to accommodate enzymes [17]. Studies on this mechanism remain insufficient. Most focus primarily on the single-molecular movement of one small section of pure cellulose fiber during a very short period of reaction time. It is difficult to get comprehensive information about the enzyme’s movement on complete cellulose during the whole hydrolytic process. Moreover, research on pure celluloses has limited reference value in the biofuel industry. It has been discovered that the reactions are strongly affected by the morphology of the substrate rather than solely by active-site considerations. Some researchers observed how small cracks evolved and increased in size during the hydrolysis process [18]. However, insufficient experimental evidence is currently available to determine the exact degradation mechanism of the pretreated cellulose.

A novel insight into the structural dynamics occurring on the pretreated cellulose and the enzyme molecules movement is necessary. There are two main difficulties: first, the heterogeneous morphology of the substrate; second, the methodological difficulties in visualizing the action of cellulases on the cellulose surface at the nanometer scale. AFM recognition imaging and single-molecule dynamic force spectroscopy (SMDFS) were applied to map the natural and pretreated plant cell wall surface and to study the affinity between noncatalytic family 3 carbohydrate-binding modules (CBM 3a) and crystalline cellulose [19]. CBM 3a has been demonstrated to specifically interact with crystalline cellulose, and has therefore been chosen as the probe to specifically recognize and map crystalline cellulose distributions [20]. In this study, we explicitly tracked individual cellulases and key, pretreated cellulose surface properties. Complete enzymatic hydrolysis of pretreated cellulose was visualized in situ and in real time by AFM topographic and recognition imaging, which provides important insight into enzyme-cellulose interactions and its limiting factors, especially those related to the pretreated substrate structure. Recognition imaging added a powerful ability to AFM to identify specific types of molecules in a compositionally complex matrix at the single-molecule level [21]. More importantly, by mapping pretreated cellulose over an entire observation area, the compositional changes over the entire complex substrate surface during a complete hydrolysis process can be determined. This paper highlights enzyme movement and how the structural changes of the pretreated cellulose during hydrolysis by CBH I and EG can reveal valuable and detailed information that is usually inaccessible by other techniques.

## Results and discussion

### Chemical composition of the pretreated biomass sample

**Fig. 1:**
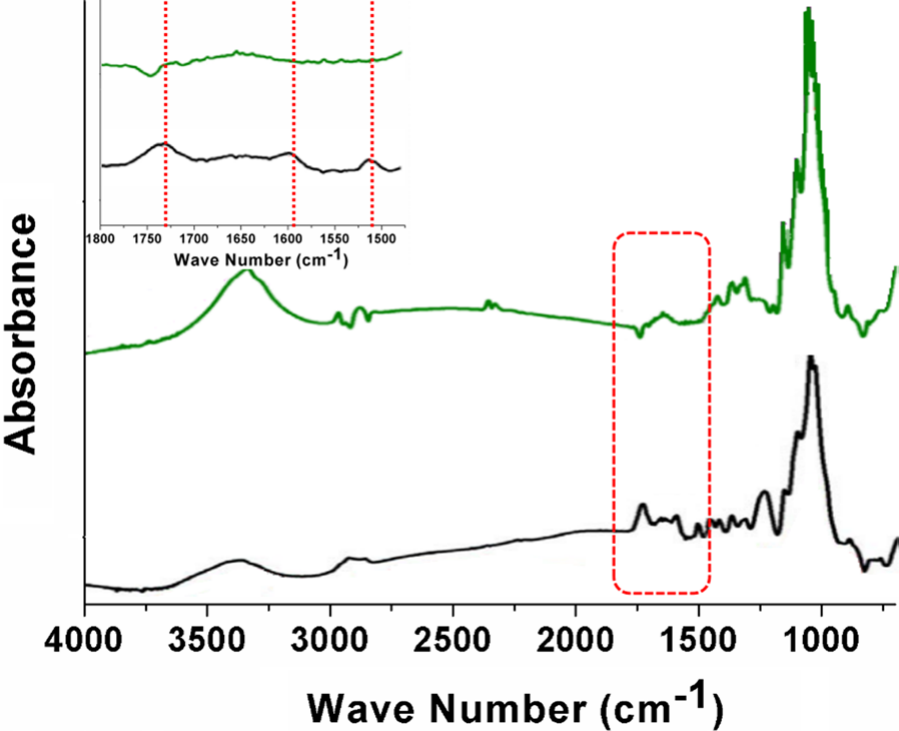
ATR-FTIR spectra of natural (*black line*) and pretreated (*green line*) poplar cell wall. Inset is the zoomed spectra from 1475 to 1800 cm^−1^

Figure 1 shows the ATR-FTIR spectra of natural (black line) and pretreated (green line) poplar cell wall from 700 to 4000 cm^−1^. The peak centered at 1732 cm^−1^ in the spectrum is attributed to the acetyl and uronic ester groups of hemicelluloses or the ester linkage of carboxylic group of ferulic and *p*-coumaric acids of lignin and/or hemicelluloses. The peaks at 1595 and 1510 cm^−1^ are ascribed to aromatic skeletal vibrations of lignin [22]. The inset from the red square highlights the surface content changes of hemicelluloses and lignin. Compared with the natural sample, the spectra of the pretreated poplar cell wall showed an obvious decrease of the peaks at 1732, 1595, and 1510 cm^−1^, indicating that, after pretreatment, the hemicelluloses and lignin on the cell wall surface almost disappeared.

### Measurement of recognition area percentage (RAP) on pretreated cellulose

**Fig. 2:**
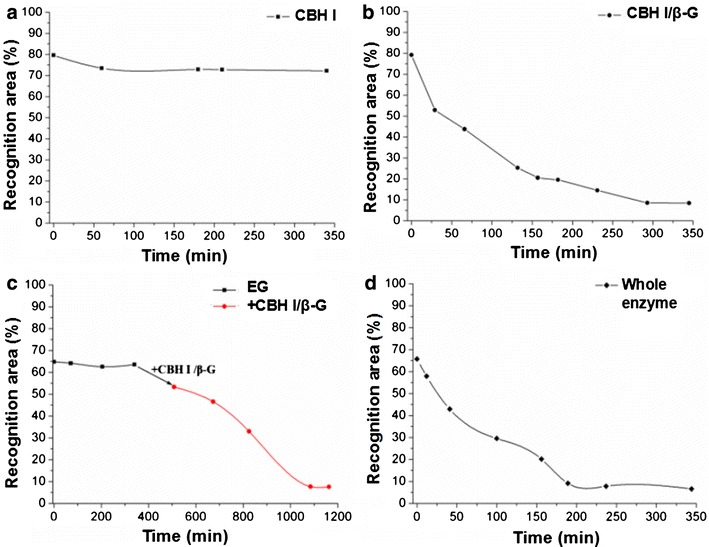
Time course of recognition area percentage (RAP) summary. Pretreated cellulose fibers hydrolyzed by CBH I (**a**), CBH I/β-G (**b**), EG and CBH I/β-G (**c**), and whole enzyme (**d**)

Having established that primarily celluloses were left behind after pretreatment successfully, RAP was calculated. RAP is the measure of percentage of exposed crystalline cellulose over the whole imaged area [23]. Details of the calculation are given in the Additional file 1: Section 2. Cellulose dispersion concentration was ~0.004 g L^−1^. Figure 2 shows RAPs changes measured under hydrolysis of CBH I, CBH I/β-G, EG and CBH I/β-G, and whole enzyme using a continuous time-lapse AFM imaging technique combining recognition imaging to follow the hydrolysis of the enzyme-cellulose systems. Additional file 1: Figure S2 (see Additional file 1: Section 2) shows the AFM topographic images and the corresponding recognition images (1 × 1 μm^2^) of cellulose hydrolyzed by EG, CBH I, CBH I/β-G, and whole enzyme, respectively. The hydrolysis of cellulose was monitored for approximately 6 h until no obvious changes could be observed. Once the RAP is determined, we calculated the digested cellulose percentages using different enzymes and the results were summarized in Table 1. The digested cellulose percentage is calculated by equation 2 shown in the Additional file 1: Section 2. For CBH I (Fig. 2a), the RAP of cellulose decreased only slightly in the first 60 min after the injection, and then kept steady for the whole imaging time of about 340 min. By 340 min, CBH I had hydrolyzed only 9.2 % of the initial cellulose, a negligible portion as highlighted by the blue circles in Additional file 1: Figure S2 (c, d, g, h). In sharp contrast, compared to the RAP values at the time of injection of CBH I and β-G (Fig. 2b), the enzyme mixture digested 89.3 % of the crystalline microfibril volume after 340 min. This value was near that of the sample incubated with the whole enzyme, which digested 89.9 % of the crystalline cellulose. Recognition imaging with CBM 3a showed few remaining cellulose fibrils, indicating that degradation of crystalline cellulose is almost complete [Additional file 1: Section 2 Figure S2(n)]. In time-resolved cellulose RAPs using CBH I/β-G, we observed that the enzymatic hydrolysis was finished at about 300 min after the enzyme injection. However, the RAP for pretreated cellulose hydrolyzed by EG is quite steady. There is a slight change in RAP due to the removal of trace amounts of amorphous cellulose. After 340 min of incubation of cellulose microfibril with EG, a mixture of CBH I and β-G was added into the sample having 65.31 % RAP and images were collected. As shown in Fig. 2c, with the injection of CBH I and β-G, degradation was renewed and RAP decreased almost linearly with time over the next 600 min. In general, 88.2 % of the cellulose was hydrolyzed after 1085-min reaction. CBH I and β-G took a much longer time to hydrolyze the crystalline cellulose after the hydrolysis by EG. The main hydrolysis process using whole enzyme (Fig. 2d) took less than 200 min. This suggests that CBH I alone is not a good option in degrading cellulose, given its incomplete hydrolysis of the substrate. Interestingly, when CBH I was injected into the liquid cell along with β-G, 55.92 % decrease of RAP was observed. These results were further confirmed by high performance anion exchange chromatography (HPAEC) of saccharides (glucose and cellobiose) from pretreated poplar cellulose hydrolyzed by CBH I and CBH I/β-G, the same samples used in the AFM imaging (Additional file 1: Section 1). Cellobiose is a disaccharide derived from the condensation of two glucose molecules linked by a β (1 → 4) bond. It is a byproduct of the enzyme-catalyzed hydrolysis of cellulose [24]. CBH I acts in a progressive manner on the reducing or non-reducing ends of cellulose polysaccharide chains, liberating either glucose or cellobiose as major products. β-glucosidases (β-G) hydrolyze soluble cellodextrins and cellobiose to glucose [25]. The HPAEC results showed no peaks that correspond to glucose or cellobiose released from the cellulose sample treated by CBH I only. As a comparison, the sample treated by CBH I/β-G showed a significant glucose peak but no cellobiose peak. No peaks are present in the control sample with cellulose alone, as expected. In the case of whole enzyme as shown in Fig. 2d, the fibrils that initially existed were dramatically degraded after the introduction of enzyme into the sample solution, leading to 89.9 % of cellulose digested.

**Table 1:** Quantitative RAPs and digested cellulose percentages of pretreated cellulose hydrolyzed by EG (+CBH I/β-G), CBH I, CBH I/β-G, and whole enzyme

EG + pretreated cellulose (65.48 %)														
Time (min)	0	71	204	340						–				
Recognition area (%)	42.47	42.02	40.96	41.59						–				
RAP (%)	64.85	64.17	62.55	63.51						–				
Digested cellulose (%)	0	1	3.5	2						–				
+CBH I/β-G														
Time (min)	–									508	673	825	1085	1163
Recognition area (%)	–									34.8	30.49	21.6	5.02	4.97
RAP (%)	–									53.26	46.56	32.98	7.66	7.59
Digested cellulose (%)	–									17.8	28.2	49.1	88.1	88.2
CBH I + pretreated cellulose (67 %)														
Time (min)	0	60	180	210	340					–				
Recognition area (%)	53.35	49.3	48.83	48.8	48.4					–				
RAP (%)	79.62	73.5	72.88	72.8	72.23					–				
Digested cellulose (%)	0	7.7	8.5	8.6	9.2					–				
CBH I/β-G + pretreated cellulose (61.32 %)														
Time (min)	0	29	66	132	157	182	231	293	345	–				
Recognition area (%)	48.56	32.42	26.84	15.51	12.6	12	8.9	5.25	5.19	–				
RAP (%)	79.19	52.87	43.77	25.29	20.54	19.56	14.51	8.56	8.46	–				
Digested cellulose (%)	0	33.2	44.7	68	74	75.3	81.7	89.2	89.3	–				
Whole enzyme + pretreated cellulose (65.65 %)														
Time (min)	0	12	41	109	156	189	237	344		–				
Recognition area (%)	43.16	38.04	28.17	19.4	13.24	6.01	5.13	4.34		–				
RAP (%)	65.74	57.94	42.9	29.55	20.16	9.15	7.81	6.61		–				
Digested cellulose (%)	0	11.8	34.7	36.19	69.3	86	88.1	89.9		–				

The total surface areas of imaged pretreated cellulose before the hydrolysis experiments were 65.48, 67, 61.32 and 65.65 %, respectively

These RAP studies indicate that CBH I alone degrades crystalline fibers extremely slowly. A sufficient amount of β-G, which hydrolyzes soluble cellodextrins and cellobiose to glucose, can prevent inhibition by the existence of cellobiose. Therefore, a higher initial cellulose hydrolysis rate was observed with the combination of CBH I and β-G. The whole enzyme contains the chain-end-cleaving cellobiohydrolases (CBH I, CBH II) and internally chain-cleaving EG as its major enzyme components. It is generally believed that CBH I molecules first bind to the planar surface of cellulose via binding module and then move along the fiber chain progressively. CBH II and EG efficiently remove amorphous cellulose and polish crystalline regions for CBH I attack. The synergetic effect between the different components in whole enzyme can accelerate the cellulose hydrolysis rate dramatically. However, the combination of CBH I and β-G exhibited a similar degradation capacity as whole enzyme. The synergetic effect of the different enzymatic components in the whole enzyme was not obvious on the extracted cellulose during the hydrolysis process. One reason is probably that the extracted sample may have removed most of the amorphous parts, the other reason maybe the cracks on the surface of the crystalline part help the effective immobilization of the enzymatic molecules effectively even without the clearing action of EG and CBH II. The hydrolysis of pretreated cellulose fibers was also performed exclusively by the EG. In theory, EG can remove the amorphous domain of cellulose to create more reducing end for the hydrolysis by CBH I. Nonetheless, the addition of CBH I and β-G to the microfibrils pretreated with EG digested the crystalline cellulose much slower than the sample treated only with CBH I and β-G in our experiment. To elucidate this phenomenon, we performed the hydrolysis experiment of EG on a pretreated cellulose including both the crystalline and amorphous parts.

The RAP studies confirm the roles of different enzymes during the hydrolysis process. For the pretreated cellulose, we can do the hydrolysis just using CBH I and β-G instead of the whole enzyme to save the cost. We also proved that the recognition experiment is an effective and reliable method to study the cellulose hydrolysis. Then we employed the AFM recognition imaging to follow the enzymatic hydrolysis process of individual cellulose to reveal the degrading mechanism at a single-molecule level.

### Real-time AFM imaging of enzymatic cellulose hydrolyzed by EG

**Fig. 3:**
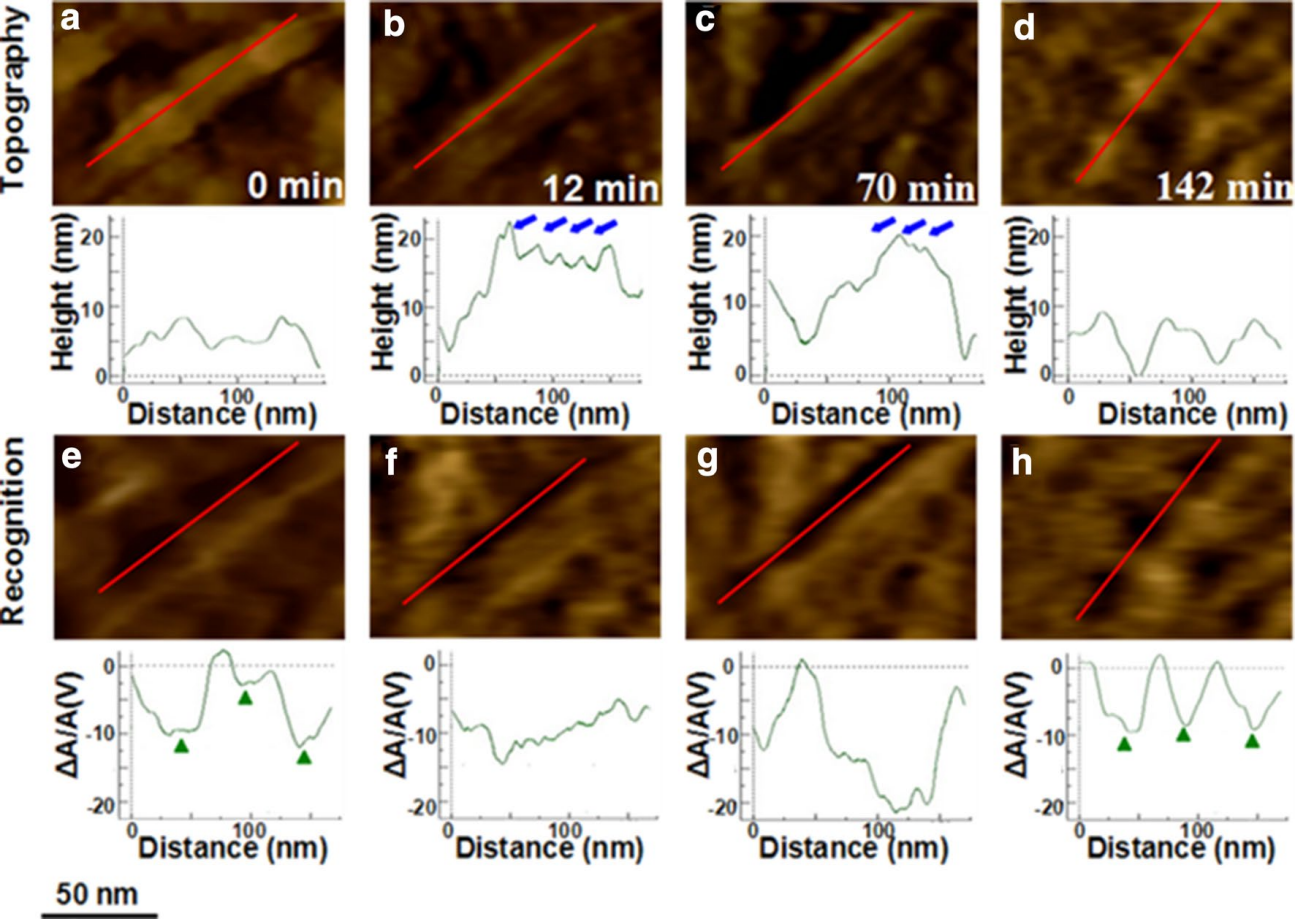
Real-time observation of pretreated poplar cellulose incubated with EG from 0 to 142 min. **a**–**d** and **e**–**h** The topographic and corresponding recognition images, respectively. The cross section analysis along the *red lines* is presented under each image. The *scale* shown at the *bottom* applies to all images

Figure 3 shows a series of time-lapse topography images and corresponding recognition images of the target cellulose microfibrils after the addition of 0.05 mU EG into the AFM liquid cell. These images are taken from Additional file 1: Figure S3. The image collected before the addition of EG (Fig. 3a) shows selected cellulose microfibrils with a width of 40 nm and a length of 175 nm. We found that this cellulose has three, discontinuous regions of crystalline cellulose recognition signal from the corresponding recognition image (Fig. 3e). The structure shown on topography images without the recognition signal is amorphous cellulose. The next three sets of images in Fig. 3 were collected at different times during the 142-min experiment after injection of EG. EG molecules bound on the surface of the cellulose led to a height increase from 5 ~ 10 to 15 ~ 20 nm. The additional EG molecules bound to the cellulose are indicated by the blue arrows shown in Fig. 3c. The size of EG molecule is expected to be about 10 nm. Abuja et al. [26] have investigated the structures of EG from *T. reesei* using small-angle x-ray scattering. The molecular size parameters: radius of gyration is 4.74 nm, overall length 18.0 nm, and diameter 5.3 nm. Considering the space conformation, we think the 10-nm size is reasonable. However, the recognition signal increased after adding EG. We think these EG molecules affect the recognition signal, but the exact mechanism remains unknown. The decrease in height is probably due to hydrolysis by EG. During hydrolysis, EG molecules appeared to be bound to amorphous cellulose with no sliding along the microfibril observed. As the experiment time progressed, the fiber structures became much easier to recognize. EG first degraded the amorphous cellulose covered on the crystalline cellulose. The length of the crystallites along the fiber directions can be obtained from the recognition images. In the following steps, EG molecules began to hydrolyze the amorphous cellulose between the crystallites. Finally, only pure crystallites can be observed on the pictures at 142 min. The last recognition image (Fig. 3h) is similar to Fig. 3e, collected before the addition of enzyme. The similar parts of crystalline cellulose were marked by green solid triangles in the cross section of Fig. 3e and h. The only slight change is the location of the crystallites because of the removal of the amorphous region.

Apparently, EG preferentially hydrolyzed the outermost amorphous parts covered on the crystalline cellulose. Therefore, the action of EG would expose pure crystalline cellulose to be hydrolyzed more easily with the progressive action of CBH I. However, from the RAP data we find that the addition of CBH I and β-G to the fiber pretreated with EG digested the crystalline cellulose much slower than the sample treated only with CBH I and β-G. From the last image at 142 min, we did not find the presence of EG non-specifically adsorbed on the fiber surface. One possibility is that the products of the EG hydrolysis inhibited the further attack by the CBH I. In the next step, we employed AFM recognition imaging to study the degradation mechanism of enzyme CBH I on crystalline cellulose.

### Real-time AFM imaging of enzymatic cellulose hydrolyzed by CBH I

Some papers have shown the hydrolysis procedure when the CBH I worked with other kinds of enzyme molecules. A number of cracks generated on the surface of the cellulose became the starting point of the movement of hydrolysis molecules. CBH I acted on the cellulose elongation direction. At the same time, there were other enzymes embedded deeply into the cracks. Our research presents a picture of how the CBH I molecules work without the help of other enzymes on the surface of the cellulose. In the following study, we mapped an integral pretreated crystalline cellulose and studied the movement of CBH I molecule along an intact cellulose fiber. In order to observe the hydrolytic activity of CBH I obviously, the combination of CBH I and β-G in Tris–Cl buffer was added into the AFM liquid cell fixed with pretreated cellulose. We also performed further analysis combining both the ragged morphology of the pretreated cellulose and the single enzyme molecule movement.

**Fig. 4:**
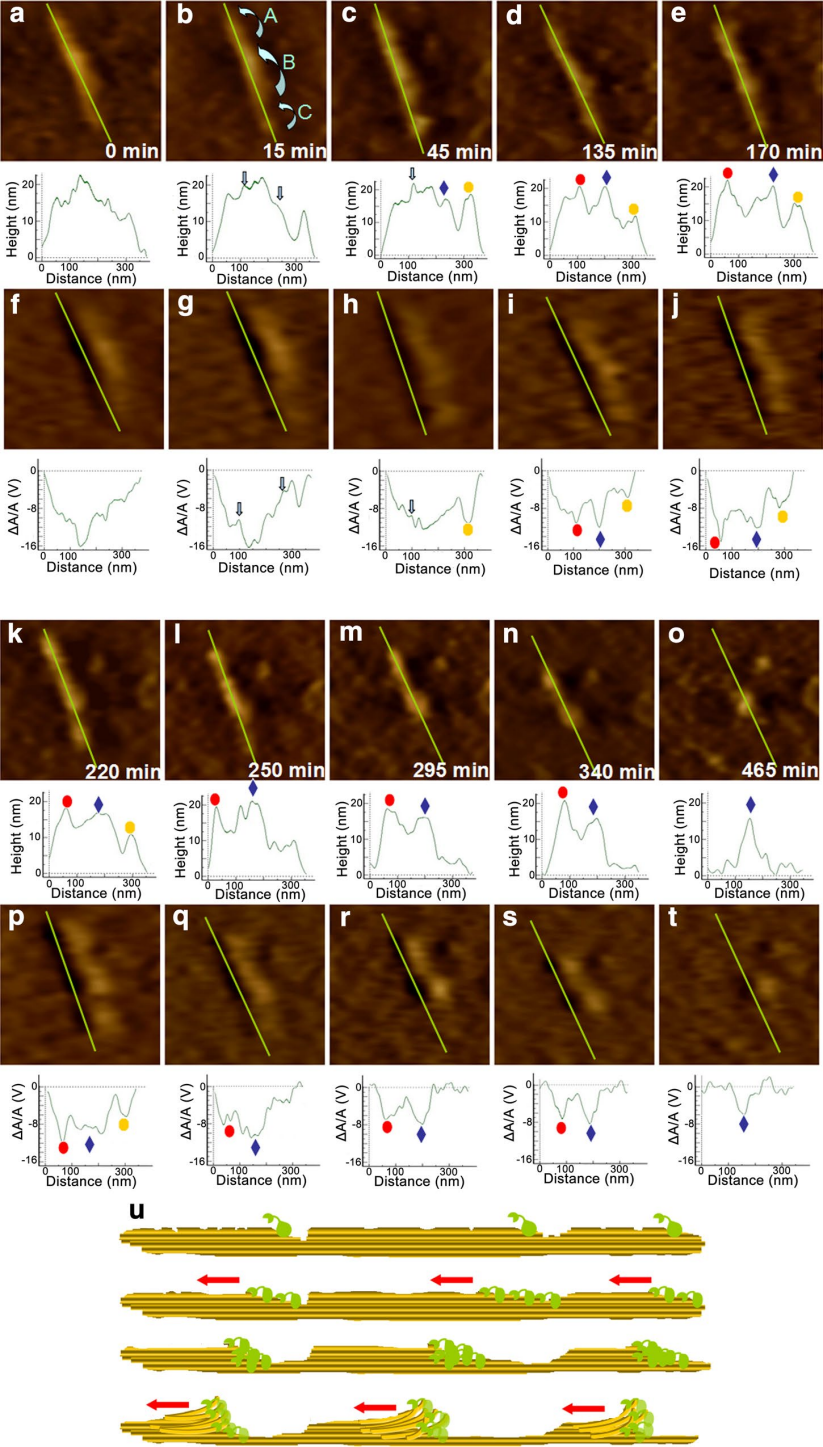
Time-lapse AFM topography and recognition images of an isolated pretreated crystalline cellulose hydrolyzed by CBH I and β-G. Topography images (**a**–**e**, **k**–**o**) and recognition images (**f**–**j**, **p**–**t**) show the cellulose structure before enzyme hydrolysis and at 15, 45, 135, 170, 220, 250, 295, 340, 465 min after addition of CBH I and β-G. The cross section analysis along the *green lines* is presented under each image. The cellulose is divided into three regions and labeled them as *A*, *B*, and *C* in (**b**). Peaks generated from the cellulose peeling action on each region are marked using *solid red circle*, *blue diamond* and *yellow circle* in the cross section profiles, separately. All images were taken in 400 × 400 nm scan size. **u** Schematic presentation of possible hydrolysis mechanism of pretreated cell wall on the presence of CBH I

Figure 4a–t illustrates the progression of enzyme action on a crystalline cellulose microfibril observed over a period of around 500 min. Figure 4a–e and k–o is topographic images and Fig. 4f–j and p–t is recognition images. The height profiles of the microfibrils taken at selected time intervals during the incubation with CBH I and β-G are also presented below each AFM image. Figure 4a shows a crystalline cellulose microfibril before the addition of enzyme. The microfibril length is about 300 nm, and its irregular profile indicates the presence of some defects caused by the pretreatment. A mixture of CBH I and β-G (0.00005 U) was then injected into the AFM liquid cell without disengaging the set point of the AFM tip. Figure 4b–e and g–t indicates the changes of the microfibril during the 500 min reaction after the enzyme injection. Enzyme molecules bound to the cellulose, increasing the height by about 4 nm after 15 min compared with the cross section before the adding of enzyme. This change in height is consistent with the CBH I molecule size as reported by Igarashi et al. [27]. Enzyme molecules were observed to move very fast, with a travel velocity of about 3 nm s^−1^, which is consistent with the velocity reported previously [16]. Cellulosic structure is typically heterogeneous and porous, consisting of both external and internal surface areas. The internal surface area, consisting of internal pores and fissures typically arises from discontinuities during the formation of the solid substrate, or from surface openings, internal slits, voids, or spaces formed during pretreatment [28]. Enzyme molecules were found to start moving from many points on the surface of the substrate. Based on all the AFM images, we found three regions in the cellulose that degraded at different rates. At first, the bottom region disappeared after 295 min (Fig. 4m). After that, the upper region was hydrolyzed completely after 465 min. We divided the cellulose into three regions and labeled them as A, B, and C as shown in Fig. 4b at 15 min. The different regions have consecutive cellulose on the bottom hydrolyzed simultaneously. But surprisingly, we did not find that the different regions were hydrolyzed synchronously, causing them to ultimately achieve the same height. We assume that, after the exfoliation of one region, new cracks were produced for the enzyme molecules to immobilize. The cross section profile of each part is irregular with several cracks. So the different regions were hydrolyzed independently.

The reaction rate of cellulose crystallites is strongly dependent on crystal size, shape and the accessibility of binding sites on crystals [29], so the larger region B was degraded slower than the smaller regions A and C. After 45 min of enzymatic hydrolysis, the cellulose microfibril frayed primarily near the cracks. The microfibril in the crack area became visibly narrower than it was initially. After 250 min, microfibrils were significantly shortened. The frayed edges of microfibril indicate that the digestion areas showed irrelevant corresponding topographic and recognition information. Therefore, the new structure on the location having the irrelevant signal was most likely CBH I molecule covering the cellulose surface. There were no CBH I molecules in the smallest region C. However, region C showed a much steeper profile. We argue that the CBH I detached from region C within 15 min. At 45 min, we were only able to label CBH I molecules that were observed in region A. Region B and C showed higher height with stronger recognition signals, and the peak location corresponded with the peak at 15 min. These additional structures must be crystalline cellulose which caused the strong recognition signal. This phenomenon is similar to the “traffic jam” that Kiyohiko Igarashi et al. [30] have proposed. In the process of crystalline cellulose hydrolysis, a single CBH I molecule was unable to climb over the higher region of cellulose and therefore halted. Then many following molecules stacked behind it on the cellulose surface, therefore leading to a “traffic jam.” However, the blockage was cleared after several subsequent molecules also blocked at the same point, and then the enzyme molecules started to move again along the surface. As a consequence, one or more layers of glycan chains on the surface of the cellulose bundle were peeled off. The small peaks appearing in the images (Fig. 4c–e, k–n) must attribute to the peeling of the glycan chains by the enzyme molecules. This behavior also reflected the increase of recognition signal in the peeled region. We marked these three regions of peaks using solid red circle, blue diamond and yellow circle in the cross section profiles (Fig. 4c–e, h–k, l–t). The hydrolysis mechanism of CBH I was schematically represented in Fig. 4u.

**Fig. 5:**
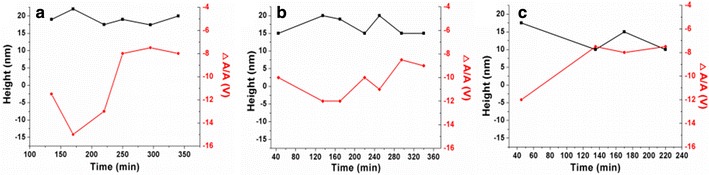
Corresponding heights (*black lines*) and recognition signals (*red lines*) changes of the generated peaks on region A (**a**), B (**b**) and C (**c**)

**Fig. 6:**
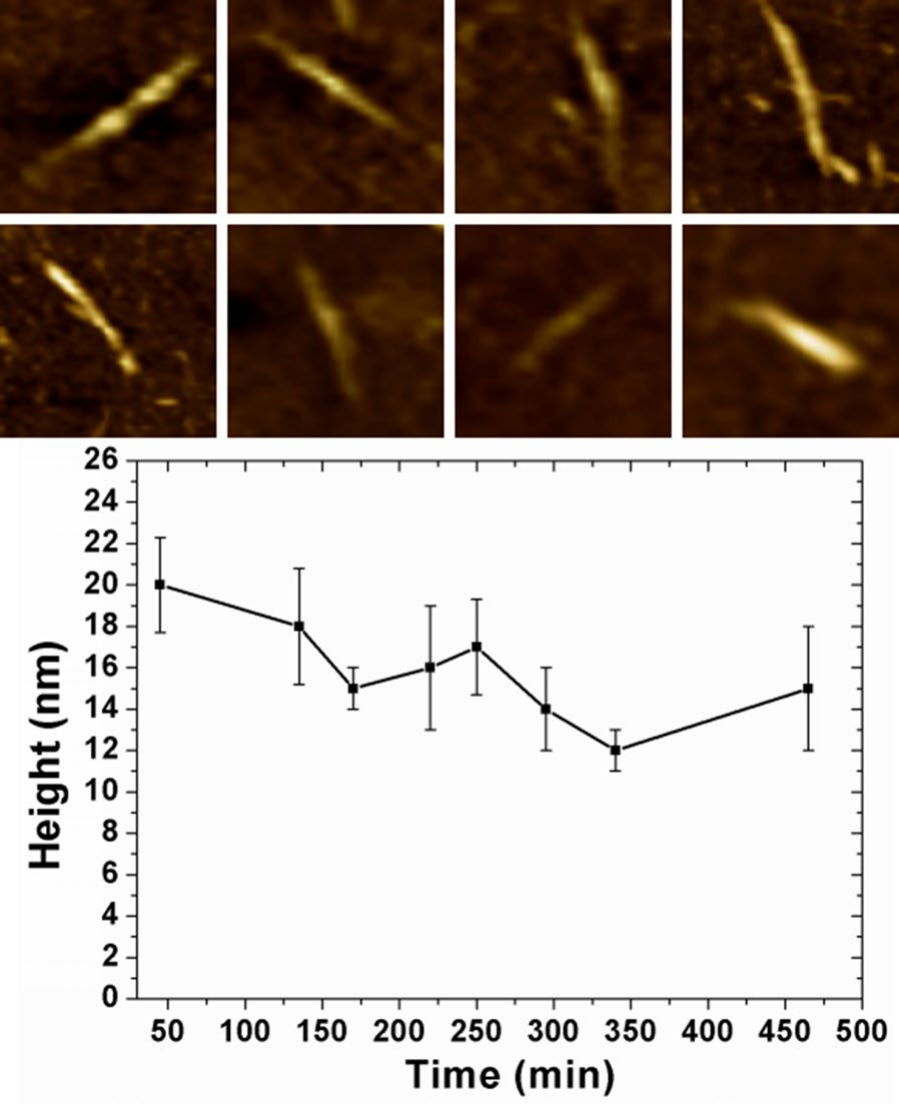
AFM topography images of eight randomly selected crystalline celluloses and statistic of their generated peaks height at 15, 45, 135, 170, 220, 250, 295, 340, and 465 min during enzymatic hydrolysis process. *Error bars* indicate standard deviation

The cross section along the green line in Fig. 4a shows that region A has more cracks. We can find many small size cracks on region A during the first 135 min. There were more enzyme molecules combined on the surface of region A because of the cracks. Therefore, when region A and region B had the similar height, the corresponding recognition signal in region A was much smaller than region B. However, there was just one jam on this area during the whole hydrolysis process. We questioned whether the jam needs a certain number of celluloses. If the crack is not big enough to initiate the hydrolysis of enough cellulose, the cellulose between two cracks will degrade smoothly without any peeling from the stopped enzymatic molecules. From 250 min, we did not find any peak which means the blocking of enzyme molecules existed in region C. This part of cellulose was progressively hydrolyzed with some cracks produced by the last peeling. This confirms that perhaps the blocking happened with a certain amount of cellulose hydrolysis. Then we collected corresponding heights and recognition signal changes of the generated peaks on region A (a), B (b) and C (c) which are defined on Fig. 4. In Fig. 5, the fluctuations in height of all these three regions are very stable. The height values for the larger region A and B are on the range from 15 to 22.5 nm. For the smaller region B, the peak height changes from 10 to 17.5 nm. We can observe that the peaks on region A and B disappeared from 465 and 250 min, separately. But they still have some cellulose fragments with very weak recognition signals shown on the images after the disappearance of the peaks. All the recognition signals of these peaks decreased to almost 8 V at the end of the hydrolysis. Then, the cellulose hydrolysis did not generate any “traffic jam,” and we cannot find any peak from the cross section profiles. The extracted crystalline cellulose microfibrils have anisotropy. In order to further confirm the precondition of the generation of the peeling, we picked eight independent crystalline cellulose microfibrils and collected the peaks information during their hydrolysis process. The AFM topography images in Fig. 6 reveal that all the eight individual crystalline microfibrils consist of incoherent regions. In the hydrolysis process as shown in Fig. 4, each region generated peaks on their cross section profiles. We did a statistical height measure of their generated peaks at 15, 45, 135, 170, 220, 250, 295, 340, and 465 min. At each time point, 16 peaks were taken randomly. The height values range from 11 to 24 nm. We assume that the “traffic jam” occurred when the peeled microfibril achieved a height above 11 nm. When the height is not large enough, the cellulose microfibril can be depolymerized to soluble sugars progressively without any stopping of enzyme molecules movement.

Above all, the peeling always happened with width enough cellulose fibers and stopped enzyme molecules. In order to achieve more efficient hydrolysis, we can try to preprocess the cellulose to get a smaller width and then the cellulose can be depolymerized progressively with less peeling. We also suppose that the “traffic jam” caused by the stopped enzyme molecules may have a relationship with the complex situation of the already peeled cellulose parts. For example, the peeled cellulose microfibrils were intertwined together, and the enzyme molecule cannot degrade the cellulose with a smooth movement along the microfibril. When a certain number of celluloses got involved into this complex situation with many jammed enzyme molecules, this block could only be peeled off. We cannot confirm these theories now. However, this is a most probable situation based on our experimental results. A much higher resolution technology is necessary for further research.

## Conclusions

Using functional AFM topographic and recognition imaging, we reported a real-time single-molecule visualization to follow the details of complete enzymatic hydrolysis process of pretreated plant cell wall cellulose using individual enzymes. The profile of pretreated cellulose was irregular with several cracks. At first, the action mode of the enzyme molecules depended on the size of the cracks. If the crack was small, the cellulose between two cracks will degrade progressively. If the crack was big enough to initiate the hydrolysis of enough number of cellulose to create the halt of enzyme molecules, glycan chains on the surface of the cellulose bundle will be peeled off. The heights of peeled microfibril were bigger than 11 nm. So the action mode also had a relationship with the width of the cellulose. After the exfoliation of one region, new cracks were reproduced for the enzyme molecules to immobilize for the adjacent cellulose part. Cellulose with a smaller width can be depolymerized progressively with less peeling. In order to achieve higher hydrolysis efficiency during biofuel production, we can try to preprocess the cellulose to get a smaller width. By recording RAP changes with various combination of enzymes, we quantitatively monitored the complete single-molecular cellulose hydrolysis process and found that the combination of CBH I and β-G exhibited similar degradation capacity as whole enzyme. This study suggests that we can use just CBH I and β-G instead of whole enzyme to hydrolyze extracted biomass, reducing the cost of pricy enzymes.

## Methods

### Pretreatment of biomass samples

The extraction of crystalline cellulose fibrils has been depicted in our previous work [23]. Briefly, 1 mg of microtomed poplar slice was treated by soaking into 1 % *w/v* NaOH (Lot: 124K00851) and Na_2_S (Lot 10H0154) (Sigma–Aldrich, St. Louis, MO) solution at 80 °C for 1.5 h. The sample was then bleached by 1.7 % *w/v* sodium chlorite (Lot: H40643, J. T. Baker, Phillipsburg, NJ) at 80 °C for 2.5 h in the presence of an acetate buffer (0.135 g NaOH and 0.375 mL glacial acetate acid (Sigma–Aldrich, St. Louis, MO) in 5 mL distilled water). The bleached cellulose fibers were centrifuged using a microcentrifuge (Model LR564958, Thermo Scientific, Waltham, MA) eight times with purified water and subsequently air-dried. Further, the fibers were hydrolyzed in 64 % *w/w* sulfuric acid (Lot: 2012032751, Sigma–Aldrich, St. Louis, MO) at 60 °C for 30 min under continuous magnetic stirring using a magnetic stirrer (Model PC-420D, Corning-PC4200, NY). The reaction was stopped by adding 2 mL cold purified water. The diluted suspension was repeatedly centrifuged at 10,000 rpm for 10 min until a turbid suspension was obtained. The suspension was collected and dialyzed using a micro dialyzer (Lot: 8854, River St. Seguin, TX) for 5 h. Finally, the suspension was sonicated using a sonicating bath (Model 3510R-MT, Branson Ultrasonics Corporation, Danbury, CT) for 30 min and stored at 4 °C with addition of 0.05 % sodium azide (Lot: BCBD9551V, Sigma–Aldrich, St. Louis, MO). The concentration of the pretreated sample was calculated as about 0.4 g L^−1^ by weight method.

### AFM tip functionalization

The AFM tips (CS-25 silicon, Lot: AP50152) with a nominal spring constant of 0.1 N m^−1^ were purchased from Nanoscience Instruments, Phoenix, AZ. The recombinant CBM 3a was provided by the Complex Carbohydrate Research Center, University of Georgia, Athens, GA. The CBM 3a-AFM tip functionalization procedure has been described in a previous publication [31]. Accurate orientation of the biomolecules was achieved using the site-directed Ni–NTA-His system. We used PEG2000 linker (Lot: JG125493, Nanocs Inc. NY) to provide enough freedom for the CBM 3a molecule to properly bind to the crystalline cellulose surface. AFM images were collected using a Pico Plus system with an Agilent multipurpose AFM scanner. All images were taken using non-contact, top magnetic AC (Top MAC) mode under the control of Pico TREC (Model N9610A, Agilent Technologies, Santa Clara, CA), the mode of which can minimize the forces applied on the sample.

### Hydrolysis experiment strategy and AFM data collection

Cellobiohydrolase I (Lot: SLBF4539, Sigma–Aldrich, St. Louis, MO), EG (Lot: S LBK0939, β-G (Lot: SLBF836ZV) and 1,4-(1,3:1,4)-β-d-Glucan 4-glucano-hydrolase whole enzyme (Lot: SLBD3796V) solution were purchased from Sigma–Aldrich Company, St. Louis, MO. Before the hydrolysis experiment, we diluted the enzyme solution to the concentration 0.0001 U using the Tris–Cl buffer. The stored extracted cellulose solution was diluted to 0.004 g L^−1^. Four micro-liters cellulose suspension was dropped onto the cleaned glass surface. After 30 min, the glass surface was washed by 0.2 mL purified water for five times to remove the unattached cellulose. After drying the cellulose sample for 6 h, the substrate remains stable in solution during the entire experiment period. Finally, the dried glass chip was fixed onto an AFM liquid cell and then filled with 0.4 mL Tris–Cl buffer (10 mM Tris–Cl and 150 mM NaCl, pH = 7.5). The AFM liquid cell was equipped with a homemade capillary port on one side for the addition of enzyme solutions for in situ imaging. All imaging trials were carried out at room temperature (20 ± 4 °C). To ensure absolute stability, the AFM was put in an acoustic and vibration isolation chamber. In each trial, target sample areas were chosen and scanned for at least 30 min before injecting the enzyme solution. After obtaining one set of high-resolution AFM topography and recognition images, the enzyme solution (0.0001 U) in 100 μL sodium acetate (Lot: 122HO7671, Sigma–Aldrich, St. Louis, MO) buffer (50 mM, pH 4.8) was gently and carefully injected into the liquid cell to ensure minimum interference to the scanning during the injection process. The hydrolysis process was then monitored in the following hours until the reaction reached equilibrium. Each AFM image was obtained under a scan speed of 6 μm s^−1^.

### Fourier transform infrared (FTIR) spectroscopy

The FTIR spectra were taken on a grazing angle attenuated total reflectance Fourier transform infrared (ATR-FTIR) instrument (Nicolet model 6700, Thermo Electron Corporation, Waltham, MA). Spectra were obtained with 4 cm^−1^ resolution at 64 scans for both the background and samples. After drying, the sample was pressed against the ATR crystal surface by a built-in pressure applicator (Harrick Scientific Products, Inc. Pleasantville, NY).

## Additional file

10.1186/s13068-016-0498-x Supporting information.

## Abbreviations

AFM: atomic force microscopy
CBM: carbohydrate-binding module
RAP: recognition area percentage
CBH I: exoglucanase
EG: endoglucanases
β-G: β-glucosidases
