# Supplementary material for: Real-time single molecular study of a pretreated cellulose hydrolysis mode and individual enzyme movement
Source: Biotechnol Biofuels. 2016 Apr 12;9:85. doi: 10.1186/s13068-016-0498-x (PMC4828794; doi:10.1186/s13068-016-0498-x)
Supplement: Supplementary file 1 — 10.1186/s13068-016-0498-x Supporting information. [file 13068_2016_498_MOESM1_ESM.pdf]

Supporting Information

Real-time single molecular study of pretreated cellulose hydrolysis mode and individual enzyme movement

Yanan Zhang[1, 2], Mengmeng Zhang[1], Robert Reese[1], Haiqian Zhang[2] and Bingqian Xu[1 *]

[1]Single Molecule Study Laboratory, College of Engineering and Nanoscale Science and Engineering Center, University of Georgia, Athens, GA 30602, USA.

[2]College of Materials Science and Technology, Nanjing University of Aeronautics and Astronautics, Nanjing, 210016, P.R.China

Corresponding author. E-mail: bxu@engr.uga.edu; Fax: +1-706-542-3804; Tel: +1-706-542-0502

1. HPAEC analysis. Cellulose enzymatic hydrolysis was carried out using 0.004% (w/w) pretreated cellulose, with loading of 0.00005U CBH I and CBH I/*β*-G, respectively. A control cellulose sample without any enzymatic molecules was also tested. The reaction system had a substrate consistency of 2% (w/w) in 0.05 M sodium citrate buffer (pH 4.8) and was placed within a shaking incubator. Hydrolysis was stopped after a time period of 1/2 h. Enzymes were then deactivated by boiling the hydrolysis system for 5 min. The control sample and the CBH I sample were diluted in half and run. The CBH I/*β*-G sample was diluted 50 times and run. The samples were analyzed by HPAEC-PAD using a Dionex ICS3000 system equipped with a gradient pump, an electrochemical detector, and an autosampler. The sample was separated using a Dionex CarboPac PA20 (3 x 150 mm) analytical column with an amino trap. Alongside these three samples, a standard solution of glucose and cellobiose was also run.

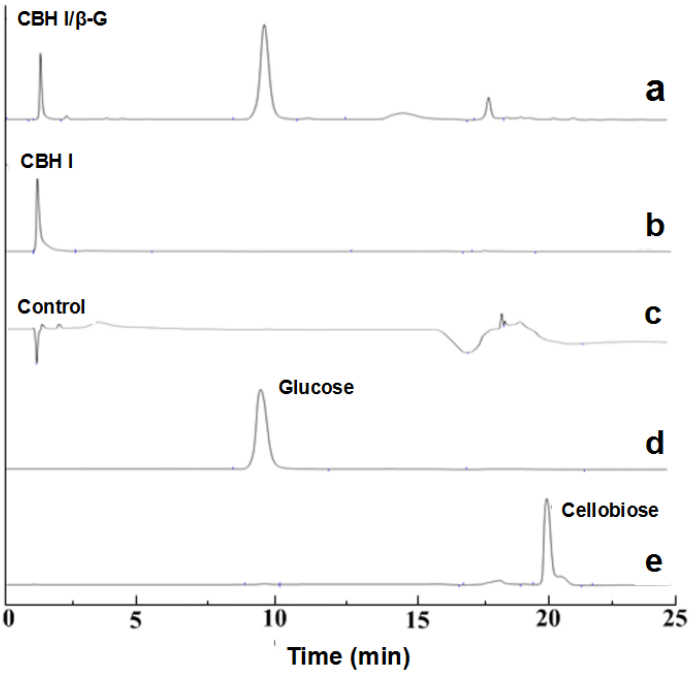

Figure S1. HPEAC/PAD profile of pretreated cellulose (c) and cellulose hydrolyzed by CBH I/ β-G (a) and CBH I (b). (d) and (e) is the profile of standard solution of glucose and cellobiose, respectively. There are no peeks that correspond to glucose or cellobiose released from the cellulose sample treated by CBH I only. The

sample treated by CBH I/β-G exhibited a significant glucose peak without cellobiose peak. No peaks are present in the control sample with cellulose alone.

2. Calculation of recognition area percentage (RAP). The calculated RAP is the measure of percentage of exposed cellulose over the whole imaged area. The method has been introduced in the supporting file of one paper we have published [1]. A single recognition image is in size of 10 μm ×10 μm. Each recognition image of different system was divided into 8 parts. Generally, the average RAP was calculated based on the relative RAP of each part.

The relative RAP of recognition was calculated using equation (1):

$$\text{Relative RAP (\%)} = \frac{\text{Recognition area on crystalline cellulose microfibrils at x min } (\text{nm}^2)}{\text{Total surface area of imaged pretreated cellulose at 0 min } (\text{nm}^2)} \quad (1)$$

The digested cellulose percentage was calculated using equation (2):

$$\text{Digested cellulose (\%)} = \frac{\text{Relative RAP at 0 min (\%) - Relative RAP at x min (\%)}}{\text{Relative RAP at 0 min (\%)}} \quad (2)$$

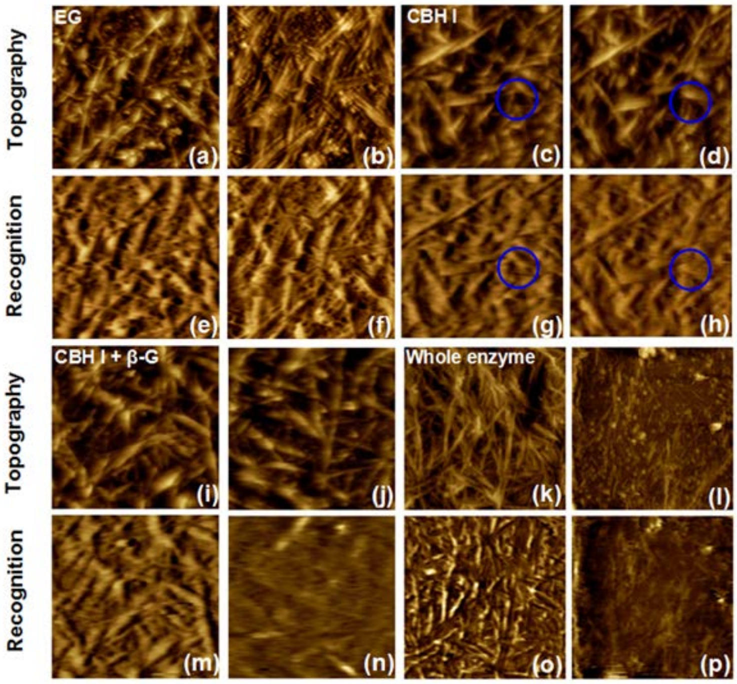

Figure S2. Representative in situ AFM topography (a-d, i-l) and recognition (e-h, m-p) images of extracted cellulose acquired before and after the injection of EG (a, b, e, f), CBH I (c, d, g, h), CBH I /β-G (i, j, m, n), and whole enzyme (k, l, o, p). The AFM images were 1μm×1μm.The blue circles highlight the fiber degradation during the reaction.

3. Real-time AFM imaging of enzymatic cellulose hydrolyzed by EG

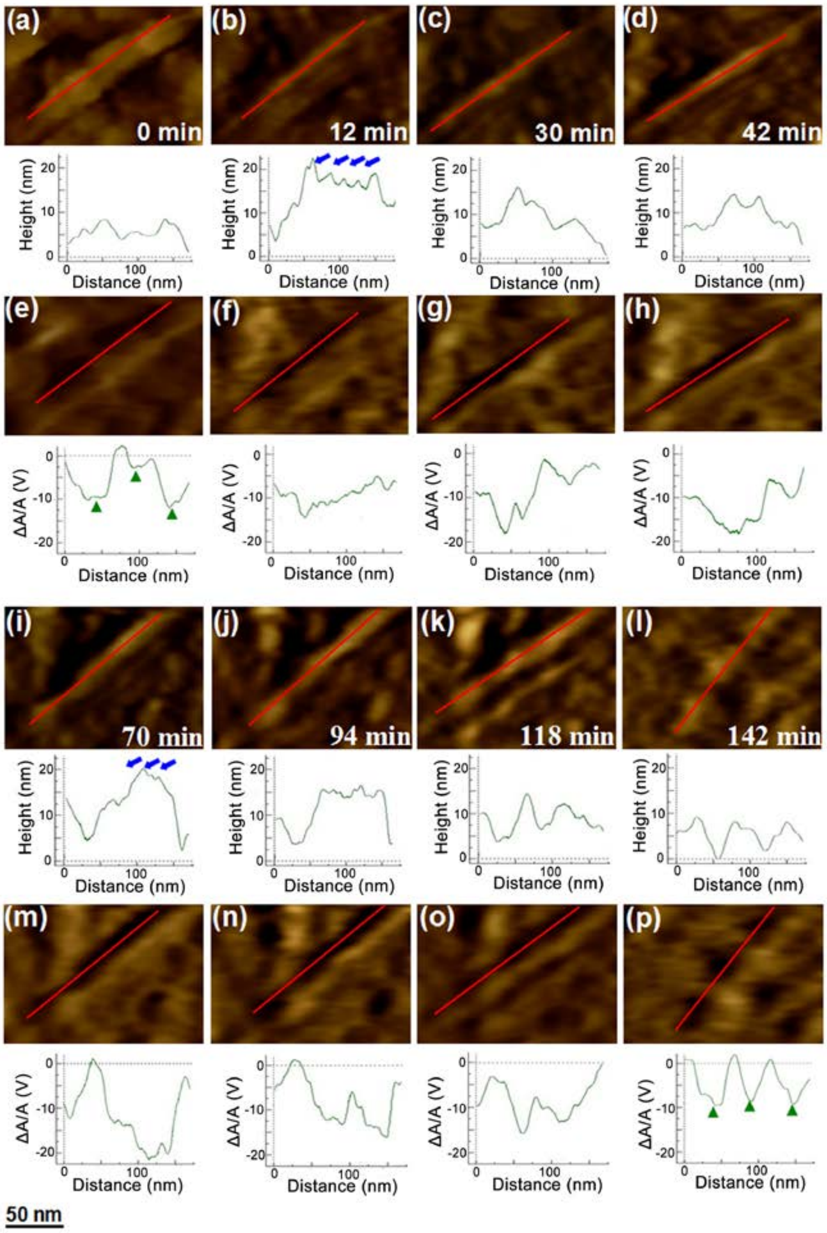

Figure S3. Real-time observation of pretreated poplar cellulose incubated with EG from 0 min to 142 min. (a-d, i-l) and (e-h, m-p): The topographic and corresponding recognition images, respectively. The cross-section analysis along the red lines is presented under each image. The scale shown at the bottom applies to all images.

1. Zhang M, Chen G, Kumar R, Xu B: **Mapping out the structural changes of natural and pretreated plant cell wall surfaces by atomic force microscopy single molecular recognition imaging.** *Biotechnol Biofuels* 2013, **6**: 147.
